# Simple and Robust Approach for Determination of Total Protein Content in Plant Samples

**DOI:** 10.3390/foods14030358

**Published:** 2025-01-22

**Authors:** Yulia S. Vershinina, Ilya V. Mitin, Andrey V. Garmay, Gleb K. Sugakov, Irina A. Veselova

**Affiliations:** 1 Chemistry Department, Lomonosov Moscow State University, Moscow 119991, Russia; ilia.mitin@chemistry.msu.ru (I.V.M.); andrew-garmay@yandex.ru (A.V.G.); irina.veselova@mail.ru (I.A.V.); 2 Institute for African Studies of the Russian Academy of Sciences, Spiridonovka Str., 30/1, Moscow 123001, Russia; g.sugakov@inafr.ru

**Keywords:** sunflower meal, plant protein, nitrogen-based methods, Lowry’s method

## Abstract

The determination of total protein in plant samples is a difficult task, as classical nitrogen-based methods are not selective for the nature of nitrogen, and the results of biochemical methods are influenced by both associated compounds and the complex composition of the protein matrix. Using electrophoretic separation of three commercial sunflower protein samples, it was determined that the studied proteins are a mixture of salt-soluble globulins and water-soluble albumins of different molecular weights. The total protein content of the studied samples was determined using five spectrophotometric methods: direct spectrophotometry, bicinchoninic acid assay, and Benedict’s, Bradford’s, and Lowry’s methods. After comparing the results obtained, it was concluded that, for the determination of protein in these plant materials, the use of the Dumas nitrogen-based method in tandem with Lowry’s spectrophotometric method is the most suitable.

## 1. Introduction

Proteins are the most important macronutrient in the human diet [1]. They play an important role in the growth and maintenance of the human body and, along with carbohydrates and lipids, are energy-giving nutrients. In addition, proteins perform a wide range of other functions in the body, such as enzymatic activity and the transport of nutrients and other biochemical compounds across cell membranes [2]. The nutritional quality of proteins depends on their bioavailability, digestibility, purity, the presence of anti-nutrient substances, and the amino acid profile. Amino acids, being the main structural compounds of proteins, participate in the formation of muscle fibers and immune system cells and help maintain many vital functions of the body [3].

The general recommendation for protein intake for adults is between 0.8 and 1.0 g per kilogram of body weight per day [4]. The human protein requirement is mainly met through nutrition, and animal products are still the main source of protein [5].

The global population is projected to reach 9.7 billion by 2050 [6]. At the same time, global demand for protein from both animal and plant sources is expected to increase. However, animal proteins are expensive in terms of both market price and environmental impact [7]. Their production requires huge land areas as well as about 100 times more water than an equivalent amount of plant protein [8].

The most efficient utilization of plant proteins will become critical when the supply of animal proteins reaches the limits of production capacity [9]. The realization of the need for food security and the challenge of an ever-growing world population are driving a shift towards a more sustainable diet, which requires less dependence on animal products and represents a huge potential for agriculture to explore alternative protein sources [3].

Currently, soy protein is leading the way as an alternative plant protein to replace animal protein. However, a number of new foods are beginning to emerge that use pea, chickpea, lupin, flax, sunflower, rapeseed, etc., as protein sources [3].

Increasing importance is being placed on reusing by-products from food waste, especially those that have the potential to contain bioactive compounds [10]. Since protein extraction from agro-industrial by-products helps to minimize waste disposal and maximize resource utilization, oilseed by-products are the most valuable [11]. In 2019–2020, global oilseed production reached 580.69 million tons, resulting in a large amount of by-products. A total of 20 million tons of these were sunflower meal [5].

Sunflower meal, formed during the process of oil extraction, has a high protein content (40–50%) that is complete in its amino acid composition and is only slightly inferior to soy protein in terms of lysine and isoleucine content (Table 1).

Among alternative plant sources of protein, sunflower is of particular interest due to its wide distribution and low cost [7]. In addition, sunflower meal protein is practically free of anti-nutrients, except for non-starch polysaccharides and phenolic compounds (Table 2).

Due to the growing interest of producers in including proteins derived from secondary products of the agro-industrial complex in human diets, there is a demand for fast and affordable methods for determining these substances. At the same time, food protein analysis is not always a simple procedure. The complexity of protein composition and interactions between different biologically active compounds of the matrix can reduce the availability of proteins, leading to an underestimation of their content [12].

The reference method for protein determination in the food industry is the Kjeldahl method. This method indirectly determines the total protein content by measuring nitrogen in a sample and then multiplying it by a conversion factor. A common conversion factor of 6.25 is used for most protein sources [13].

Another method for determining total nitrogen is the automated Dumas method. Unlike the Kjeldahl method, it is faster and safer. However, both nitrogen-based methods have a common disadvantage: the methods are non-selective in terms of the nature of the nitrogen in the analyzed samples and determine the total amount of the nitrogen, whether it is organic and integrated into protein molecules or added to the product as an extraneous nitrogen-containing substance. A number of substances, such as melamine, ammonium sulfate, and urea, give the same results as the Kjeldahl and Dumas methods for proteins and can be used as falsified adulterants [14].

An alternative to the determination of total nitrogen is spectrophotometric methods, which are based both on the absorption by the structures of aromatic rings in certain amino acids and on the interaction between dyes or copper ions and charged amino acid residues or peptide bonds [15]. These methods are widely used in the analysis of biological fluids, and there are sporadic and not always successful results of their validation in plant samples [16,17,18]. When they are used for protein determination in sunflower meal isolates, the influence of not only associated compounds (polysaccharides, phenolic compounds, etc.) but also the complex composition of the protein matrix is taken into account. The protein matrix consists of a mixture of two different types of proteins: water-soluble albumins and salt-soluble globulins with different molecular weights [7].

The aim of this work is a comparative study of nitrogen-based and biochemical methods, as well as the development of a simple and robust approach for determining the protein content of plant samples with complex matrices, using sunflower meal as an example.

## 2. Materials and Methods

### 2.1. Preparation of Reagents and Working Solutions

A total of 0.1 M NaOH solution was prepared and used for dissolving BSA and protein samples.

Benedict’s and Bradford’s reagents, working solutions for protein determination using the Lowry’s method and with bicinchoninic acid (BCA), were prepared according to the methods described in Table 3.

Deionized water with a resistivity of at least 18.2 MΩ cm (“Millipore”, Guyancourt, France) was used to prepare all aqueous solutions.

### 2.2. Sample Preparation

Three commercial samples of sunflower meal protein were used in this study. The claimed protein content of sample-1 is 90%, sample-2 is 82.5%, and sample-3 is 78%. Bovine serum albumin (99%, “Sigma-Aldrich”, Waltham, MA, USA) was used as a protein standard in all analyses.

Stock standard solutions of bovine serum albumin (BSA) and three commercial samples of sunflower meal protein (SMP) were prepared by dissolving the respective suspensions in an alkaline solution (0.1 M NaOH). The sample solutions were stirred for 10 min using a Multi-RS-60 rotary stirrer (“BIOSAN”, Riga, Latvia). The precipitate was separated by centrifugation using an Eppendorf Centrifuge 5910 R (“Eppendorf”, Hamburg, Germany) at 15,900 RPM for 10 min. After that, the supernatant was separated from the precipitate.

### 2.3. Protein Electrophoresis in Polyacrylamide Gel

Electrophoresis was performed on vertical gel plates (9 cm × 12 cm × 0.1 cm) in discrete mode under conditions of a linear gradient of polyacrylamide. The concentrating gel was polymerized in 120 mM Tris-HCl, pH 6.8, with 0.1% sodium dodecyl sulfate (SDS). The separating gel was polymerized in 0.375 M Tris-HCl, pH 8.8, with 0.1% SDS. Before electrophoresis, the samples were boiled at 60 °C for 3 min in a 60 mM Tris-HCl buffer (pH 6.8), 2% SDS, 10% glycerol, 5% 2-mercaptoethanol, and 0.001% bromophenol blue.

Electrophoresis was performed using 25 mM Tris, 0.19 M glycine, and 0.01% SDS as electrode buffer at a constant current of 20 mA prior to protein incorporation into the separating gel and at 50 mA afterwards.

After electrophoresis, the gel was fixed in a solution of 5% perchloric acid and 50% methanol for 20 min at 55 °C and washed with water. Protein bands were stained with a 0.04% solution of Coomassie G-250 in 3.4% perchloric acid at 30 °C for 3 min. Unbound dye was removed by washing with water at 90 °C.

### 2.4. Nitrogen-Based and Biochemical Methods for Protein Assay

The Dumas method [24] was used to determine the content of total nitrogen. In a Perkin Elmer 2400 Series II CHNS/O Elemental Analyzer (“PerkinElmer”, Shelton, CT, USA), samples with a known mass were burned at 900 °C in the presence of oxygen. The protein content was obtained by multiplying the total nitrogen content by a standard conversion factor of 6.25 [25].

Spectrophotometric analysis was performed using a SPECTROstar Nano plate reader (“BMG Labtech”, Ortenberg, Germany) and a 96-well quartz plate.

**Direct spectrophotometric method (280 nm).** Protein assay was determined according to the method [26]. To plot the standard calibration curve, a series of standard solutions with protein concentrations ranging from 200 to 2000 μg/mL were prepared from the stock standard solution of BSA. A series of test solutions were prepared at concentrations of 800, 1200, and 1600 μg/mL for each SMP sample. The absorbance of the solutions was measured at a wavelength of 280 nm.

**Benedict’s method (microbiuret method).** The protein assay was performed according to the method adapted for a 96-well plate [19]. To plot the standard calibration curve, a series of standard solutions with protein concentrations ranging from 200 to 2000 μg/mL were prepared from the stock standard solution of BSA. A series of test solutions were prepared at concentrations of 800, 1200, and 1600 μg/mL for each SMP sample. A total of 10 μL of Benedict’s reagent and 150 μL of 3% sodium hydroxide solution were added to 50 μL of each solution. The mixture was incubated for 15 min at room temperature, and the absorbance was measured at a wavelength of 300 nm.

**BCA method.** The protein assay was performed according to the method [20]. To plot the standard calibration curve, a series of standard solutions with protein concentrations ranging from 200 to 2000 μg/mL were prepared from the stock standard solution of BSA. A series of test solutions were prepared at concentrations of 800, 1200, and 1600 μg/mL for each SMP sample. A total of 200 μL of the working solution was added to 25 μL of each solution, mixed on a vibration shaker for 30 s, and then incubated for 30 min at 37 °C. The absorbance was measured at a wavelength of 562 nm.

**Bradford’s method.** Protein assay was performed according to the method described by Bradford [21], adapted to a 96-well plate. To plot the standard calibration curve, a series of standard solutions with protein concentrations ranging from 10 to 100 μg/mL were prepared from the stock standard solution of BSA. A series of test solutions were prepared at concentrations of 40, 60, and 80 μg/mL for each SMP sample. A total of 150 μL of Bradford’s reagent was added to 150 μL of each solution and incubated for 5 min at room temperature. The absorbance was measured at a wavelength of 595 nm.

**Lowry’s method.** Protein assay was performed according to the method presented by Lowry et al. [22] except that sodium tartrate was replaced by sodium citrate [23]. To plot the standard calibration curve, a series of standard solutions with protein concentrations ranging from 10 to 100 μg/mL were prepared from the stock standard solution of BSA. A series of test solutions were prepared at concentrations of 40, 60, and 80 μg/mL for each SMP sample. A total of 200 μL of the working solution was added to 40 μL of each solution and incubated for 10 min at room temperature. Then 20 μL of 1 M Folin–Ciocalteu reagent was added, mixed, and incubated for 30 min at room temperature. The absorbance was measured at a wavelength of 760 nm.

### 2.5. Statistical Analysis

At least three independent analyses (*n* = 3) of the protein content in the samples were carried out. The “OriginPro 2018” software was used to analyze the data using mathematical statistics methods.

## 3. Results and Discussion

### 3.1. Electrophoresis

**Figure 1:**
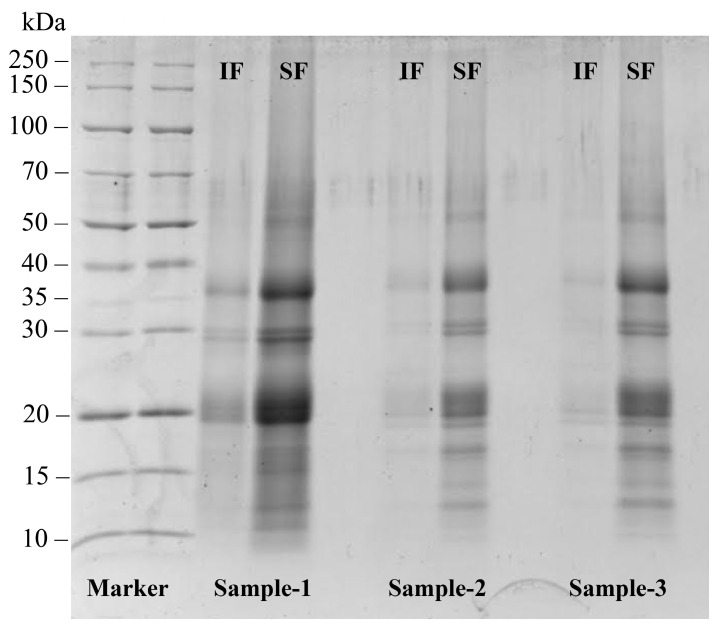
SDS-PAGE electrophoresis of soluble (SF) and insoluble (IF) fractions of three commercial SMP samples.

The fractional composition of soluble and insoluble fractions of the three commercial SMP samples was studied using electrophoresis (Figure 1). The results showed that protein fragments were present in both fractions and peptide bands were similar in all three protein samples.

Based on the data obtained, it can be stated that all commercial samples contain low molecular weight proteins (10–20 kDa), which correspond to 2S albumins, and proteins with a molecular mass of 30–50 kDa, which correspond to dissociated 11S globulins.

### 3.2. Protein Assay

The Kjeldahl method is a classical method for protein analysis in the food industry based on the determination of total nitrogen and conversion to protein content using appropriate coefficients. However, this method is difficult to perform and takes several hours and requires the use of hazardous chemicals.

In addition, the Dumas method is also used. In the first stage, a sample is burned in an oxygen atmosphere at about 1000 °C to produce water, carbon dioxide, nitrogen, and nitrogen oxides. The nitrogen oxides are then reduced to elemental nitrogen on the copper surface, and water and carbon dioxide are separated. In the last step, a thermal conductivity detector is used to determine the nitrogen content of the sample. A common disadvantage of nitrogen-based methods is that they determine nitrogen regardless of whether it is organic, part of the protein molecule, or introduced into a product with an extraneous nitrogen-containing substance.

Biochemical methods of determination are an alternative, but they are broadly applicable for the determination of individual protein forms. Difficulties of determination arise when moving to plant samples that contain different proteins, both in nature and in terms of molecular weight, and are characterized by a complex matrix composition.

Since each of the protein determination methods described in Table 4 has its own drawbacks, all five methods were used to determine the protein content of three commercial samples.

**Figure 2:**
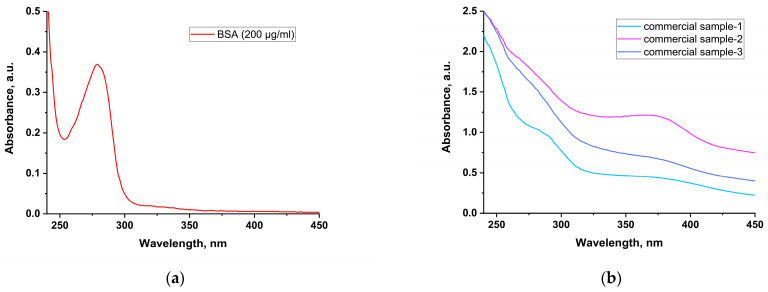
UV spectra of (**a**) standard solution of BSA (200 μg/mL) and (**b**) tested solutions of three commercial SMP samples.

Direct spectrophotometric determination of protein at 280 nm was not possible because all three samples showed no clear peak in this part of the spectrum. This may be due to the different amino acid composition of BSA and sunflower protein. Another reason may be the presence of interfering matrix components, such as chlorogenic acid with an absorption maximum at 330 nm (Figure 2).

**Figure 3:**
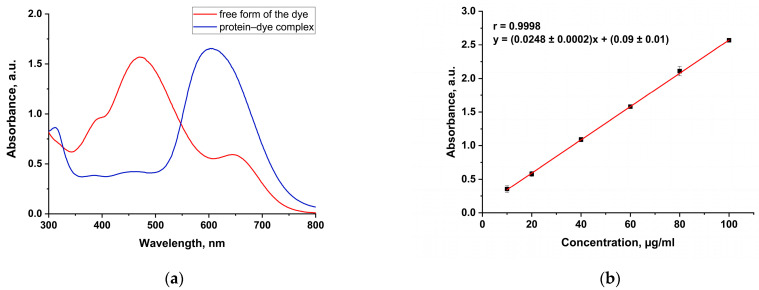
(**a**) UV spectra of the free and bound forms of the dye; (**b**) calibration curve for the Bradford’s method.

In protein determination by the Bradford’s method, the nature of the protein standard is crucial. The BSA standard used in this study had an excessive binding response to Coomassie and thus underestimated the protein content of all three commercial samples (Figure 3).

**Figure 4:**
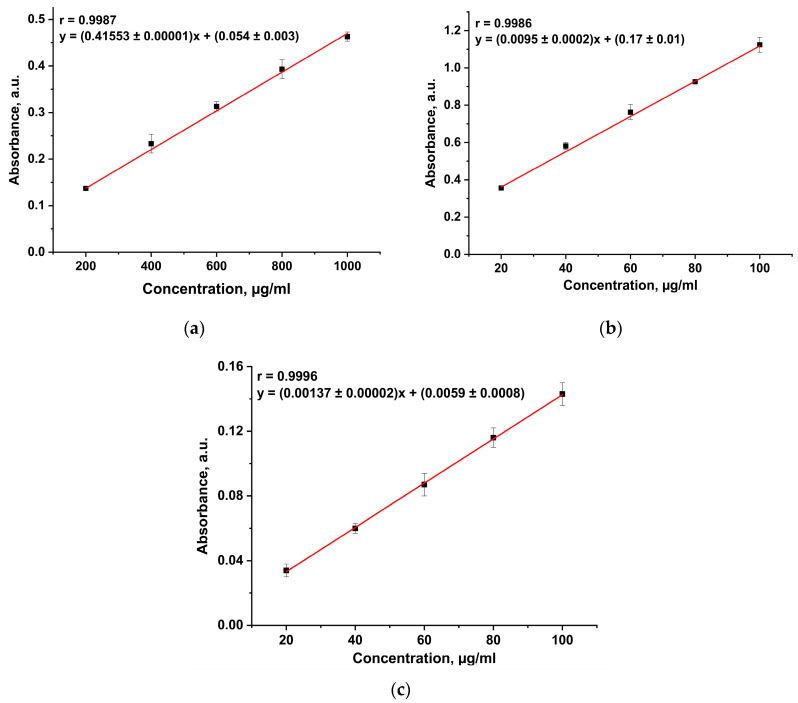
Calibration curve for the (**a**) Benedict’s method, (**b**) BCA method, and (**c**) Lowry’s method.

Calibration curves with a correlation coefficient of at least 0.998 were obtained for the Benedict’s, BCA, and Lowry’s methods (Figure 4). However, using the Benedict’s and BCA methods, the results obtained for all three commercial protein samples were underestimated compared to those reported by manufacturers. This could be explained by the interaction between the associated components of the protein matrix and copper ions or by the influence of reducing agents.

Table 5 shows the results of the protein content of SMP samples determined using nitrogen-based and biochemical methods.

The protein content results for commercial sample-1 and commercial sample-3 determined by the Dumas method were consistent with the manufacturer’s claims and agreed with the results obtained using the Lowry’s method. In addition, the results obtained by these two methods are consistent for commercial sample-2, but they differ from the protein content declared by the manufacturer. This does not exclude the possibility of falsification.

## 4. Conclusions

In the course of this study, it was found that the most promising approach to determining the total amount of protein is based on using the Dumas nitrogen-based analysis method in tandem with the Lowry’s spectroscopic method, which relies on the peptide bonding reactions. At the same time, the high sensitivity of the Lowry’s method allows for the exclusion of interfering influences from matrix components (polysaccharides and phenolic compounds).

## Figures and Tables

**Table 1:** The amino acid composition of sunflower meal protein compared to “ideal” protein [3].

Essential Amino Acids	Content, g/100 g Protein	
	«Ideal» Protein	Sunflower Meal Protein
Histidine	1.5	2.6
Isoleucine	3.0	5.0
Leucine	5.9	6.8
Lysine	4.5	4.4
Threonine	2.3	4.0
Tryptophan	0.6	-
Valine	3.9	5.4
Methionine	2.2	5.1
Phenylalanine + tyrosine	3.8	8.9

**Table 2:** Composition of sunflower meal [7].

Component	Content, %
Proteins	20–40
Carbohydrates	4–18
Lipids	47–65
Fatty acids:	
-palmitic acid	5–7
-stearic acid	2–6
-arachidonic acid	0–0.3
-oleic acid	15–37
-linoleic acid	51–73
-linolenic acid	<0.3
Tocopherol	0.07
Carotenoids	0.01–0.02
Vitamin B1	0.002
Chlorogenic acid	0.5–2.8
Quinic acid	0.12–0.25
Caffeic acid	0.05–0.29
Minerals	3–4
Potassium	0.67–0.75
Phosphorus	0.60–0.94
Sulfur	0.26–0.32
Magnesium	0.35–0.41
Calcium	0.08–0.10
Sodium	0.02

**Table 3:** Summary of reagent preparation methods used for the determination of proteins by the spectrophotometric method.

Method	Composition and Methods of Reagent Preparation	References
Benedict’s method	**Reagent A:** A total of 17.3 g of sodium citrate and 10 g of sodium carbonate were dissolved in 40 mL of water. **Reagent B:** A total of 1.73 g of copper sulfate was dissolved in 10 mL of water. **Working solution:** Reagent A and reagent B were mixed and diluted with more water to make a final volume of 100 mL.	[19]
BCA method	**Reagent A:** A total of 0.1 g of sodium bicinchoninate, 2 g of sodium carbonate, 0.16 g of sodium tartrate, and 0.4 g of sodium hydroxide were mixed with 0.95 g of sodium bicarbonate and diluted in water to obtain a final volume of 100 mL. **Reagent B:** A total of 0.4 g of copper sulfate was dissolved in 10 mL of water. **Working solution:** A total of 1 mL of reagent B was mixed with 50 mL of reagent A.	[20]
Bradford’s method	A total of 10 mg of Coomassie Brilliant Blue G-250 dye was dissolved in 5 mL of 95% ethanol. A total of 10 mL of 85% phosphoric acid was added, and the solution was diluted with water to obtain a final volume of 100 mL.	[21]
Lowry’s method	**Reagent A:** A total of 2 g of sodium carbonate was dissolved in 100 mL of 0.1 M sodium hydroxide solution. **Reagent B:** A total of 0.025 g of copper sulfate was dissolved in 50 mL of 1% sodium citrate solution. **Working solution:** A total of 1 mL of reagent B was mixed with 50 mL of reagent A.	[22,23]

**Table 4:** Brief description of biochemical methods for protein determination and their disadvantages.

Method	Range of Concentrations	Method Principle	Disadvantages
Direct spectrophotometric method	20–2000 μg/mL	This method is based on the ability of aromatic amino acids (tryptophan, tyrosine, and, to a lesser extent, phenylalanine) to absorb ultraviolet (UV) light at 280 nm.	Since proteins differ in the content of aromatic amino acids, their absorption in the UV region of the spectrum varies greatly. When real samples are analyzed, an amino acid peak may be overlapped by an interfering matrix compound with an absorption maximum in the same region of the spectrum.
Benedict’s method	200–2000 μg/mL	This method is based on the interaction of copper ions Cu^2+^ with peptide bonds of protein in an alkaline medium, forming a violet-colored complex.	This method is not recommended for reactions in solutions containing ammonium salts, due to the possibility of complex formation with copper, as well as in turbid or precipitated solutions.
BCA method	200–2000 μg/mL	This method is based on reducing Cu^2+^ to Cu^+^ by interacting with the peptide bond of a protein and forming a colored Cu^+^ complex with bicinchoninic acid.	Determination is hindered by the presence of reducing substances, such as sugars, ascorbic acid, and thiol compounds.
Bradford’s method	10–100 μg/mL	This method is based on the binding of Coomassie Blue G250 dye to amino acid residues in protein (primarily arginine and lysine), which shifts the absorption maximum from 470 nm (free dye) to 595 nm (bound dye), at which point the determination is carried out.	The dye most strongly binds to arginine and lysine residues, which can lead to errors in the determination of different types of proteins. BSA has an excessive dye-binding response and thus may underestimate the protein content of the analyzed sample.
Lowry’s method	10–100 μg/mL	This method is based on the formation of colored products from aromatic amino acids using the Folin–Ciocalteu reagent in combination with the biuret reaction to detect peptide bonds.	Phenolic compounds, sugars, thiol compounds, and reducing agents interfere with analysis by enhancing background or leveling coloration with proteins.

**Table 5:** Protein content of SMP samples.

Sample	Bradford’s Method	Benedict’s Method	BCA Method	Lowry’s Method	Dumas Method
Commercial sample-1 (90%)	63.4 ± 1.7	91.0 ± 1.8	87.5 ± 3.3	89.7 ± 2.4	90.50 ± 0.07
Commercial sample-2 (82.5%)	44.2 ± 3.9	54.5 ± 5.3	75.3 ± 3.5	54.5 ± 3.3	51.56 ± 0.01
Commercial sample-3 (78%)	58.6 ± 1.1	62.2 ± 1.4	63.1 ± 1.3	76.1 ± 1.7	81.56 ± 0.01

## Data Availability

All data supporting the reported results of this study are available from the corresponding author upon reasonable request.
